# Elevation of serum plasminogen activator inhibitor-1 predicts postoperative delirium independent of neural damage: a sequential analysis

**DOI:** 10.1038/s41598-022-21682-7

**Published:** 2022-10-12

**Authors:** Kazuhito Mietani, Maiko Hasegawa-Moriyama, Koichi Yagi, Reo Inoue, Toru Ogata, Nobutake Shimojo, Yasuyuki Seto, Kanji Uchida, Masahiko Sumitani

**Affiliations:** 1 Department of Anesthesiology and Pain Relief Center, The University of Tokyo Hospital, Tokyo, Japan; 2 Department of Pain and Palliative Medical Sciences, Faculty of Medicine, The University of Tokyo, 7-3-1 Hongo, Bunkyo-ku, Tokyo 113-8655 Japan; 3 Department of Gastrointestinal Surgery, The University of Tokyo Hospital, Tokyo, Japan; 4 Department of Rehabilitation Medicine, The University of Tokyo Hospital, Tokyo, Japan; 5 Department of Emergency and Critical Care Medicine, Tsukuba University Hospital, Ibaraki, Japan; 6 Department of Pain and Palliative Medicine, The University of Tokyo Hospital, Tokyo, Japan

**Keywords:** Neuroscience, Biomarkers

## Abstract

Older adult surgical patients are susceptible to developing delirium. Early intervention can be initiated if a potential biomarker associated with delirium can be identified during the acute phase of surgery. Therefore, we investigated the changes in the levels of serum inflammatory mediators responsible for delirium. Serum biomarkers were measured preoperatively to postoperative day 3 in 96 patients who underwent esophageal cancer surgery and compared between patients who did and did not develop delirium. Serum concentrations of the brain-derived phosphorylated neurofilament heavy subunit remained at higher levels throughout the entire perioperative period in patients with delirium (n = 15) than in those without delirium (n = 81). The interaction between delirium and non-delirium was significant for plasminogen activator inhibitor-1 (including age as a covariate, F = 13.360, *p* < 0.0001, η^2^_p_ = 0.134, observed power 1.000) during the perioperative periods. Plasminogen activator inhibitor-1 level discriminated between patients with and without clinically diagnosed delirium with significantly high accuracy (area under curve, 0.864; sensitivity, 1.00: negative predictive value, 1.000; *p* = 0.002). Rapid increases in the levels of serum plasminogen activator inhibitor-1 may enable clinicians to identify patients at risk of developing postoperative delirium and initiate early prevention and intervention.

## Introduction

Postoperative delirium (PD) is a common complication that occurs in 11% to 51% of surgical patients aged over 65 years^1^. Moreover, it is associated with increased length of stay and 30-day readmission^2^, a higher incidence of morbidity and mortality and increased costs during the 1-year postadmission period^3^. Despite the need for actions focused on cognitive and delirium screening, non-pharmacologic interventions, pain control, and avoidance of antipsychotics, PD is often poorly managed^4^. It has been shown that male sex, depressive symptoms, mild cognitive impairment and laboratory abnormalities are also associated with an increased risk of PD. Among the precipitating factors, the use of sedative hypnotic and anticholinergic agents, surgery, anesthesia, a high pain level, anemia, infections, acute illness and acute exacerbation of chronic illness are the most commonly reported^5^. To screen for delirium, the Confusion Assessment Method for the intensive care unit (CAM-ICU)^6^ and the Intensive Care Delirium Screening Checklist (ICDSC)^7^ are typically used in critically ill patients in the ICU. However, predicting PD remain difficult, and delayed and inaccurate diagnoses can lead to brain atrophy, even in patients who recover from PD^8^.

On the basis of a previous report that showed that surgical patients had higher rates of brain atrophy than non-surgical controls during the first follow-up interval (5–9 months)^9^, we measured the serum concentration of phosphorylated neurofilament heavy subunit (pNF-H), a major cytoskeletal protein of central nervous system (CNS) axons, in surgical patients^10,11^. Although pNF-H is not detectable in the blood of healthy patients, it is detected in 56.1 to 65.2% of surgical patients who experience PD, which suggests that pNF-H is related to PD development and delirium-related CNS damage. In addition, we recently reported that pNF-H is detected preoperatively in surgical patients at higher levels in patients with PD than in those without PD^12^. Taken together, we hypothesized that perioperative changes in pNF-H concentration reflect the vulnerability for surgery-induced cognitive decline and are a predictive factor for the severity of PD. However, changes in pNF-H concentration during the perioperative period have not been investigated to date.

In addition, significant concentrations of pro- and anti-inflammatory markers such as interleukin-6 (IL-6) are detectable in the serum and cerebrospinal fluid after surgery^13,14^, and anesthesia may modulate immune signaling pathways^15,16^. Furthermore, management during surgery and the ICU can improve cognitive outcomes. We previously reported that the elevation of the platelet endothelial cell adhesion molecule (PECAM)-1 is associated with increased serum pNF-H levels and that P-selectin is the only independent variable associated with pNF-H detection^10^. Therefore, the mediators involved in inflammation and coagulation/fibrinolysis such as PECAM-1 and P-selectin may exacerbate the disruption of the blood–brain barrier (BBB), resulting in neural tissue damage. It has been reported that increases in matrix metalloprotease (MMP)-9 and plasminogen activator inhibitor (PAI)-1 disrupt the integrity of the BBB, which results in the neurodegenerative progression of normally aging individuals and hospitalized older adult patients with PD^17,18^. In our recent study using the same dataset^12^, we found that preoperative IL-6 concentration was significantly higher whereas the concentrations of PAI-1 was significantly lower in patients with postoperative delirium. However, as well as a predictor, an objective indicator other than CAM-ICU and ICDSC might be required in clinical situations, even after delirium is suspected after surgery in the ICU. Therefore, in this study, we focused on the timing and duration of serum markers availability for the diagnosis of delirium after surgery during the perioperative periods. We first investigated whether sequential changes in the PD-related neuronal marker, pNF-H, correlate with the incidence of PD. We subsequently explored time course changes in the levels of the factors involved in inflammation and coagulation/fibrinolysis and their association with PD.

## Results

### Perioperative risk factors for delirium

As we previously reported using the same dataset^12^, PD occurred in 15 of 96 patients (15.6%) during the first 3 days following surgery (online Supporting Information S1). No patients included in this study regularly used either sedative hypnotic or anticholinergic agents before surgery. A comparison of patient’s characteristics and perioperative parameters between patients with and without PD was previously performed using the Wilcoxon rank-sum test or Pearson’s chi-squared test (online Supporting Information Table S1)^12^.

### Changes in plasma levels of pNF-H in the perioperative periods

**Figure 1:**
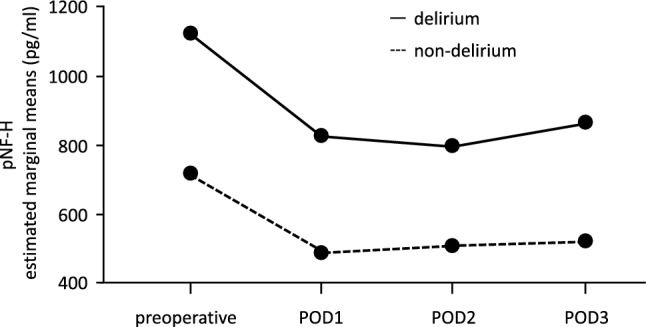
Changes in the serum levels of pNF-H in patients with (solid line) and without (dotted line) delirium. The preoperative value of serum pNF-H originated from the same dataset published in Ref.^12^. The sample size at each point is indicated in online Supporting Information Table S2. POD, postoperative day; pNF-H, phosphorylated neurofilament heavy subunit.

We previously found that preoperative levels of pNF-H were significantly higher in PD patients compared with non-PD patients^12^. In this study, changes in the levels of pNF-H throughout the perioperative periods were followed up (Fig. 1, Online Supporting Information Table S2 and S3). Because we previously found that the specificity of PD diagnosis increased when age was combined with the detection of preoperative pNF-H^12^, it was included in the analysis as a covariate. Plasma levels of pNF-H after surgery remained at a higher level in PD patients compared with non-PD patients, not only preoperatively, but also throughout the perioperative periods (Fig. 1, online Supporting Information Table S2). The interaction between PD and non-PD patients was not significant (no covariate, F = 0.536, *p* = 0.658, η^2^_p_ = 0.006, observed power = 0.159; including age as a covariate, F = 0.226, *p* = 0.879, η^2^_p_ = 0.003, observed power = 0.092) (online Supporting Information Table S3). Consistent with these results, changes in pNF-H levels were not associated with the development of PD during any period of the 3 days following surgery (Table 1). Notwithstanding, the positivity of pNF-H was significantly higher in patients with delirium than in those without delirium at all points of the perioperative periods (online Supporting Information Table S4).

**Table 1:** Logistic regression analysis for prediction of postoperative delirium.

Biomarkers	POD	Univariable OR	95% CI	*P*-value
pNF-H	1 − 0	0.90	0.75 − 1.09	0.2297
	2 − 1	0.98	0.53 − 1.73	0.9583
	3 − 2	0.89	0.50 − 1.35	0.6287
PAI-1	1 − 0	5.18	2.06 − 16.51	0.0003
	2 − 1	0.44	0.08 − 2.51	0.3337
	3 − 2	0.52	0.11 − 2.28	0.3775
MMP-9	1 − 0	0.98	0.76 − 1.40	0.9016
	2 − 1	0.81	0.68 − 0.97	0.0156
	3 − 2	1.34	1.10 − 1.88	0.0031
P-selectin	1 − 0	1.66	0.61 − 4.48	0.3047
	2 − 1	0.28	0.07 − 0.95	0.0415
	3 − 2	3.44	1.14 − 10.42	0.0157
PECAM-1	1 − 0	1.28	0.52 − 3.34	0.6015
	2 − 1	1.12	0.37 − 3.53	0.8406
	3 − 2	1.22	0.45 − 3.72	0.700
IL-6	1 − 0	0.86	0.57 − 1.33	0.4930
	2 − 1	0.92	0.47 − 1.79	0.8059
	3 − 2	1.30	0.55 − 2.88	0.5372

Difference in the log-transformed levels of each biomarker between indicated postoperative days were calculated using subtraction. 1 − 0, subtraction of preoperative pNF-H values from those on postoperative day 1; 2 − 1, subtraction of values on postoperative day 1 from those on postoperative day 2; 3 − 2, subtraction of values on postoperative day 2 from those on postoperative day 3.

Abbreviations: *POD* postoperative day; *OR* odds ratio; *CI* confidence interval; *pNF-H* phosphorylated neurofilament heavy chain; *PAI-1* plasminogen activator inhibitor-1; *MMP-9* matrix metalloproteinase-9; *PECAM-1* platelet endothelial cell adhesion molecule-1; *IL-6* interleukin-6.

Sample size at each point is indicated in online Supporting Information Table S2.

Preoperative (POD0) values of PAI-1, MMP-9, P-selectin, PECAM-1 and IL-6 come from the dataset published in Ref.^12^.

### Changes in plasma markers related to inflammation and fibrinolysis in the perioperative periods

**Figure 2:**
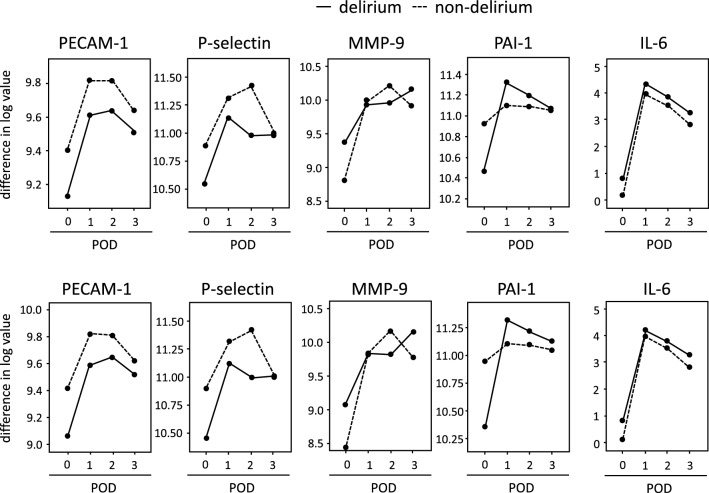
Changes in the serum levels of potential biomarkers for delirium. Preoperative (POD 0) values of PAI-1, MMP-9, P-selectin, PECAM-1, and IL-6 originated from the same dataset published in Ref.^12^. Delirium, solid line; dotted line, delirium. Upper panels, no covariate; lower panels, age as a covariate. Sample size at each point is indicated in online Supporting Information Table S2. POD, postoperative day; pNF-H, phosphorylated neurofilament heavy subunit; PAI-1, plasminogen activator inhibitor-1; MMP-9, matrix metalloproteinase-9; PECAM-1, platelet endothelial cell adhesion molecule-1; IL-6, interleukin-6.

We previously found that preoperative levels of several markers related to inflammation and fibrinolysis were different between PD and non-PD patients. To clarify the difference in time-course changes in these markers between PD and non-PD patients, the levels of these markers were measured during the perioperative periods (Fig. 2, and online Supporting information Table S2). Age was included in the analysis as a covariate (Fig. 2 and online Supporting Information Table S3).

The interaction between PD and non-PD was significant for PAI-1 (no covariate, F = 9.437, *p* < 0.0001, η^2^_p_ = 0.098, observed power 0.997; including age as a covariate, F = 13.360, *p* < 0.0001, η^2^_p_ = 0.134, observed power 1.000) during the perioperative periods (online Supporting Information Table S3. Post-hoc analysis revealed that preoperative levels of PAI-1 were significantly lower in patients with PD than in those without PD (no covariate, *p* = 0.006; including age as a covariate, *p* = 0.001). Changes in PAI-1 levels from pre-surgery to postoperative day 1 were associated with the development of PD (Univariable odds ratio = 5.18, 95% confidence interval = 2.06 − 16.51, *p* = 0.001) (Table 1). The interaction between PD and non-PD was significant for MMP-9 (no covariate, F = 4.407, p = 0.005, η^2^_p_ = 0.072, observed power 0.869; including age as a covariate, F = 4.023, p = 0.009, η^2^_p_ = 0.067, observed power 0.833) (Fig. 2, online Supporting Information Table S3). The difference in MMP-9 levels at any time point was not significant by post-hoc analysis, although changes in MMP-9 levels from postoperative days 1 to 2 and from days 2 to 3 were associated with the development of PD (Table 1). There was no significant interaction between PD and non-PD for the levels of P-selectin, PECAM-1, and IL-6, for which the observed power was < 0.8 (online Supporting Information Table S3), although changes in P-selectin levels from postoperative days 1 to 2 and from days 2 to 3 were associated with the development of PD (Table 1).

### Diagnostic accuracy of plasma PAI-1 levels for delirium

**Figure 3:**
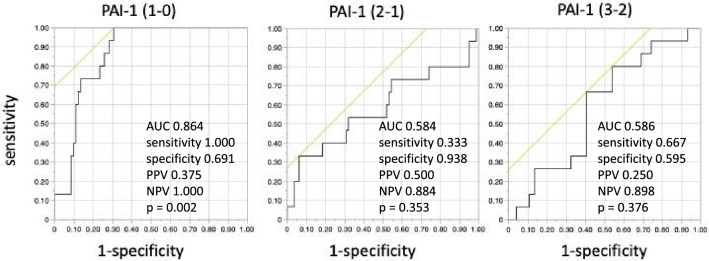
Receiver operating characteristic curve for predicting delirium by plasminogen activator inhibitor-1. Differences in the log-transformed levels of each biomarker between the indicated postoperative days were calculated using subtraction. 1 − 0, subtraction of preoperative PAI-1 values from those on postoperative day 1; 2 − 1, subtraction of values on postoperative day 1 from those on postoperative day 2; 3 − 2, subtraction of values on postoperative day 2 from those on postoperative day 3. Sample size at each point is indicated in online Supporting Information Table S2. Preoperative (POD 0) values of PAI-1 are identical to the dataset published in Ref.^12^. PAI-1, plasminogen activator inhibitor-1; AUC, area under curve; PPV, positive predictive value; NPV, negative predictive value.

Because the interaction between PD and non-PD was significant for PAI-1 with an observed power of 1.000 during the perioperative periods (online Supporting Information Table S3), and the changes in PAI-1 levels from pre-surgery to postoperative day 1 was associated with the development of PD (Table 1), ROC analysis was performed to investigate the diagnostic accuracy of PAI-1 for delirium at three time points from pre-surgery to postoperative day 3. The changes in PAI-1 levels from pre-surgery to postoperative day 1 discriminated between patients with and without PD with significantly high accuracy (area under the curve, 0.864, sensitivity, 1.000; specificity, 0.691; positive predictive value, 0.375; negative predictive value, 1.000; *p* = 0.002; Fig. 3).

## Discussion

Our recent report, in which we analyzed the dataset of the same participants during the preoperative period only, demonstrated that the detection of serum pNF-H levels before surgery is significantly associated with the incidence of PD in ICU patients following esophageal cancer surgery^12^. In the present study, sustained elevation and positivity of pNF-H concentration was detected in PD patients compared with non-PD patients during the entire perioperative period (Fig. 1 and online Supporting Information Tables S2, S3 and S4). In addition, the transient elevation of PAI-1 levels from pre-surgery to postoperative day 1 was associated with the development of PD (Fig. 2 and Table 1). Especially, the accuracy of plasma PAI-1 levels from pre-surgery to postoperative day 1 for the diagnosis of delirium was significant with high sensitivity and negative predictive value (Fig. 3).

First, the serum pNF-H level remained at a higher level in PD patients compared with non-PD patients, regardless of the occurrence of surgical events from pre-surgery to postoperative day 3 (Fig. 1 and online Supporting Information Table S2). We previously reported that an increased level of pNF-H was observed in 28.8% of patients who underwent chemotherapy but not in chemotherapy-naïve patients with early breast cancer^19^. However, neoadjuvant therapy was not associated with the incidence of delirium (online Supporting Information Table S1). These results suggest that CNS vulnerability to surgery-induced inflammation and fibrinolysis might develop before surgery, although there is no direct association between chemotherapy and the incidence of delirium. It is possible that inflammatory mediators disrupt neural function without changes in pNF-H degradation during the acute phase, or CNS atrophy may develop long-term following discharge from the ICU, as previously reported^9^.

Second, in contrast to the pNF-H level, compared with non-PD patients, the level of PAI-1, a marker of microvascular fibrinolysis, was elevated on postoperative day 1 in patients with PD. In this study, the interaction of MMP-9 was significant, although its levels at each time point was not significant (Fig. 2 and online Supporting Information Table S3). In addition, the difference in MMP-9 levels at any time point was not significant by post-hoc analysis, although changes in MMP-9 levels from postoperative day 1 to 2 and from days 2 to 3 were associated with the development of PD (Table 1). Because activation of the plasminogen activator system provokes the production of MMP-9, which leads to matrix protein degradation and BBB leakage^20^, these mediators might cooperatively deteriorate neural cognitive functions through BBB disruption.

Ballweg et al. demonstrated that a transient increase in tau is observed in patients with delirium after surgery^21^. Furthermore, plasma PAI-1 levels are elevated in patients with mild cognitive impairment and during the early stage of Alzheimer disease^22^, and increased PAI-1 expression has been observed near amyloid deposits or sites with inflammatory responses in the brains of patients with AD^23^. These results suggest that PAI-1 is involved in CNS inflammation and neural damage during both the acute and chronic phases of the neurodegenerative process.

It has been reported that concentrations of plasma PAI-1, samples of which were obtained a maximum of 72 h after organ failure, were consistently associated with ICU-related delirium, with higher PAI-1 concentrations associated with fewer delirium/coma-free days in the total cohort and longer delirium duration among survivors^24^. Our study demonstrated that during the perioperative course, only the change in serum PAI-1 levels from pre-surgery to postoperative day 1 discriminated between patients with and without PD with significantly high accuracy (Fig. 3). Taken together, the PAI-1 level at a more acute period immediately following surgery has higher sensitivity for the diagnosis of PD during the acute postoperative period. However, a direct link between pNF-H and PAI-1 was not detected (data not shown). Because pNF-H is a neuron-specific cytoskeletal protein responsible for protecting neurofilaments from degeneration^25^, the possibility that changes in serum pNF-H levels may not reflect ongoing neuronal functional changes during the perioperative period cannot be excluded. In addition, it is unclear whether the elevated serum PAI-1 levels induce neurodegeneration or whether they are elevated in quick response to prior neuronal damage. Although there are multiple reports showing an association between increased levels of PAI-1 and neurodegenerative disorders, PAI-1 is also known to prevent disintegration of neuronal networks by promoting neuroprotective signaling via the mitogen-activated protein kinase/extracellular signal-regulated kinase pathway^26^. Therefore, the role of PAI-1 in the CNS during the perioperative period should be further investigated.

IL-6 and PAI-1 are biomarkers that are not only suitable for identifying older adults at risk of frailty, but are also independently associated with a long duration of delirium in patients without dementia who are admitted to the emergency room^18^. More recently, it was reported that higher levels of preoperative IL-6 were associated with PD^27^. We recently reported that preoperative levels of IL-6 are significantly higher in patients with PD than in those without PD and are associated with PD^12^. In this study, we found that perioperative changes in IL-6 were not associated with PD (Fig. 2, Table 1, online Supporting Information Table S3). Similarly, we previously reported that P-selectin is an independent factor in the detection of pNF-H^10^. However, perioperative changes in P-selectin levels were not associated with PD, although the statistical power was not sufficient to reach a conclusion (online Supporting Information Table S3). The possibility that multiple events, such as postoperative infection (another risk factor for PD^1^) heterogeneously influence on inflammatory changes cannot be excluded.

For perioperative conditions, the differences in preoperative opioid use and surgical procedure were statistically significant (online Supporting Information Table S1)^12^. However, because the number of patients with delirium was small, these were not included in the statistical analysis as a covariate in this study. In addition, the use of epidural anesthesia was statistically significant (online Supporting Information Table S1)^12^. It has been recently reported that delirium was significantly less common in the combined epidural–general anesthesia group (1.8%) than in the general anesthesia group (5.0%), although the incidence of intraoperative hypotension (systolic blood pressure less than 80 mmHg) was higher in patients undergoing combined epidural–general anesthesia^28^, implying that direct effects of local anesthesia on the CNS rather than hypotension can be associated with delirium. In our study, epidural anesthesia was not combined in two patients developing delirium (online Supporting Information Table S1)^12^, which is consistent with the previous report^28^. In this study, local anesthetic was intraoperatively administered to all the patients in which epidural anesthesia was combined (*n* = 94). Although it was not statistically significant, the lowest mean blood pressure during anesthesia was slightly lower in patients with delirium than in those without delirium (*p* = 0.0566). There was no patient whose lowest mean blood pressure was > 60 mmHg (data not shown). In only one patient who did not develop delirium, lowest systolic blood pressure was > 80 mmHg (data not shown). The association of combined epidural anesthesia with general anesthesia and intraoperative hypotension with the incidence of delirium should be further investigated.

This study has two limitations. First, the observed power in repeated two-way analysis of variance of several markers including pNF-H, PECAM-1, P-selectin, and IL-6 was statistically low (online Supporting Information Table S3). A further large study is required for the sequential time course analysis of these markers. Second, the type and severity of PD were not evaluated in this study. Therefore, whether the changes in serum mediators differ depending on the clinical PD symptoms should be further investigated.

In conclusion, the expression of pNF-H persists at higher levels in patients with PD than in those without PD during the acute postoperative period. In contrast, the level of PAI-1 transiently increases immediately after surgery in PD patients. Therefore, the early evaluation and detection of PAI-1 as well as pNF-H may allow the prediction of PD during the acute postoperative period.

## Methods

### Ethics

The study was approved by the ethics committee of The University of Tokyo (approval ID: 11,261) and was conducted from October 2016 to June 2019. Written informed consent was obtained from each patient. The study was registered in the University Medical Information Network (UMIN trial ID: UMIN000010329). All methods were performed in accordance with the relevant guidelines and regulations.

### Study population

This was a prospective observational study conducted at The University of Tokyo Hospital^12^. We screened a total of 120 patients who underwent elective esophageal cancer surgery and provided written informed consent for participation. The population of this study was identical to that in our previous study, in which we focused on preoperative serum measurement data^12^. We obtained measurements from the preoperative to postoperative period. The sample size was determined in accordance with a previous study that showed an increase in postoperative pNF-H concentration on postoperative day 3^10^. Assuming that the baseline pNF-H level would not be detectable, a population of 96 patients would provide 90% power (p = 0.025) to show a difference in pNF-H concentration of 134 pg ml^−1^ (SD, 367 pg ml − ^1^). We used their enrolled patients’ stored serum samples. Details of the data, including the exclusion criteria, patient demographics, and pNF-H positivity, were previously described^12^. Briefly, patients scheduled to undergo esophageal cancer surgery were eligible for inclusion in the study. Surgical procedures were open, robot-assisted or mediastinoscopy-assisted esophagectomy. Exclusion criteria were as follows: (1) patients with a score of 4 on the American Society of Anesthesiologists physical classification; (2) patients with a clinically relevant cognitive dysfunction or a diagnosis of a neurological disorder before surgery; and (3) patients who were regularly prescribed tranquilizers that could influence PD^12^. According to these criteria, 24 patients were excluded.

### Patient assessment

During the first 3 days after surgery, delirium-associated symptoms were screened by the attending nurses at least three times per day using CAM-ICU during regular ward rounds. Patients with suspected PD underwent further assessment using the ICDSC to confirm the diagnosis of PD. The onset of PD was evaluated from the day of surgery to postoperative day 3.

### Measurement of samples

Blood samples were collected in EDTA-containing tubes from an arterial blood pressure monitoring line immediately after the induction of anesthesia and before the start of surgery, and were then sent for cytokine multiplex assay. Measurement of pNF-H was performed using an enzyme-linked immunosorbent assay (BioVendor, Brno, Czech Republic) according to the manufacturer’s protocol (Supplementary Material); the threshold concentration for detection was 70.5 pg/mL. Neither repeatability nor intermediate precision of internal quality control was evaluated. PECAM-1, MMP-9, PAI-1, and IL-6 were measured using a multiplex immunoassay (Luminex Assay Human Premixed Multi-Analyte Kit; R&D, Rockville, MD, USA) according to the manufacturer’s protocol (Supplementary Material). All samples were measured in duplicate.

### Statistical analysis

Analyses based on the log-transformed concentration of potential candidate biomarkers were performed as previously reported^12^. Patient characteristics were compared using Pearson’s chi-squared test or Wilcoxon rank-sum test. Comparisons of time course changes in the levels of pNF-H and inflammatory markers between patients with and without delirium were evaluated using two-way analysis of variance with and without a covariate, followed by post-hoc analysis performed with Bonferroni test. In cases in which data were missing even for one time point, all the data of these patients throughout pre-surgery to postoperative day 3 were not used for two-way analysis of variance. The sample size of each marker at each time point is described in online Supporting Information Table S2. Logistic regression analysis of the changes in potential candidate variables was performed to identify biomarkers for PD. Receiver operating characteristics curve analysis was performed to investigate whether PAI-1 levels could discriminate between patients with and without PD. *p* ≤ 0.05 was considered significant. Statistical analyses were performed using JMP Pro software version 16 (SAS Institute, Cary, NC, USA) and SPSS software version 22 (IBM Corp, Armonk, NY, USA).

## Supplementary Information

Supplementary Information 1.

Supplementary Information 2.

Supplementary Information 3.

Supplementary Information 4.

Supplementary Information 5.

## Data Availability

Anonymized data from this study are available for academic purposes upon reasonable request. Enquiries can be directed to the corresponding author.
