# Supplementary material for: Elevation of serum plasminogen activator inhibitor-1 predicts postoperative delirium independent of neural damage: a sequential analysis
Source: Sci Rep. 2022 Oct 12;12:17091. doi: 10.1038/s41598-022-21682-7 (PMC9556513; doi:10.1038/s41598-022-21682-7)
Supplement: Supplementary file 1 — Supplementary Information 1. [file 41598_2022_21682_MOESM1_ESM.docx]

**Supporting information Table S1** Comparison of patient characteristics and perioperative events between patients with and without delirium

|  | Delirium  (n = 15) | Non-delirium (n = 81) | *p* value |
| --- | --- | --- | --- |
| Age (y) | 74 ± 7 | 67 ± 9 | 0.0021 |
| Sex (male/female) | 13/2 | 69/12 | 0.8813 |
| ASA-PS (1/2/3) | 0/8/7 | 14/45/22 | 0.1226 |
| Body mass index | 21.4 ± 2.8 | 21.9 ± 3.0 | 0.5624 |
| Neoadjuvant therapy |  |  |  |
| Chemotherapy (+/-) | 12/3 | 51/30 | 0.2019 |
| Radiation (+/-) | 3/12 | 8/73 | 0.2582 |
| Preoperative opioid use (+/-) | 3/12 | 1/80 | 0.0008 |
| Surgical procedure  (open/ robot-assisted/ mediastinoscopic) | 13/1/1 | 33/35/13 | 0.0044 |
| Anesthesia type (Des/Sev/Prop) | 5/9/1 | 30/42/9 | 0.8002 |
| Anesthesia time (min) | 466 ± 109 | 495 ± 95 | 0.3409 |
| Operation time (min) | 400 ± 88 | 426 ± 97 | 0.3212 |
| Blood loss (mL) | 352 ± 282 | 488 ± 310 | 0.1389 |
| The use of epidural anesthesia (Yes/No) | 13/2 | 81/0 | 0.0009 |
| Lowest mean blood pressure during anesthesia | 44 (40-48) | 47 (44-52) | 0.0566 |

ASA-PS, American Society of Anesthesiologists physical status; Des, desflurane; Sev, sevoflurane; Prop, propofol. Data are shown as mean ± SD, number, or median (interquartile range).

All the data except lowest blood pressure during anesthesia in Table S1 were reported in Ref. 12.
