# Supplementary material for: Elevation of serum plasminogen activator inhibitor-1 predicts postoperative delirium independent of neural damage: a sequential analysis
Source: Sci Rep. 2022 Oct 12;12:17091. doi: 10.1038/s41598-022-21682-7 (PMC9556513; doi:10.1038/s41598-022-21682-7)
Supplement: Supplementary file 2 — Supplementary Information 2. [file 41598_2022_21682_MOESM2_ESM.docx]

**Supporting Information Table S2** Time course of biomarker levels in delirium and non-delirium patients.

|  | Non-delirium | | | | Delirium | | | |
| --- | --- | --- | --- | --- | --- | --- | --- | --- |
|  | Pre | POD1 | POD2 | POD3 | Pre | POD1 | POD2 | POD3 |
| pNF-H  (pg/ml) | 0  (0–668.5)  [n=80] | 0  (0–469)  [n=81] | 0  (0–413.5)  [n=81] | 0  (0–491)  [n=75] | 659  (93.8–2110)  [n=15] | 590  (145–1520)  [n=15] | 802  (307–1210)  [n=15] | 820  (258–1280)  [n=15] |
| PAI-1  (pg/ml) | 58443 (40640–74269)  [n=81] | 69750 (52072–84491)  [n=81] | 64443 (52048–80851)  [n=81] | 63599 (47503–82736)  [n=74] | 42593 (29135–67697)  [n=15] | 99524 (58961–110181)  [n=15] | 71240 (57242–117813)  [n=15] | 66076 (47426–90996)  [n=15] |
| MMP-9  (pg/ml) | 7846  (3448–15547)  [n=80] | 27567 (14885–39162)  [n=73] | 33496 (17974–59277)  [n=59] | 27182 (17963–42191)  [n=67] | 10588  (3294–16678)  [n=15] | 26398 (14630–41059)  [n=15] | 23868 (12281–35236)  [n=12] | 34502 (15654–47773)  [n=15] |
| P-selectin (pg/ml) | 50794 (32962–84491)  [n=81] | 81113 (61820–105237)  [n=81] | 101447 (69149–121504)  [n=81] | 68961 (36142–94837)  [n=74] | 33535 (23363–83905)  [n=15] | 83930 (66044–92579)  [n=15] | 63498  (43830 –79915)  [n=15] | 70494 (47350–91587)  [n=15] |
| PECAM-1  (pg/ml) | 12535  (7842–20545)  [n=81] | 18610  (14923–22955)  [n=81] | 19972  (15120–23850)  [n=81] | 17743  (11406–23392)  [n=74] | 8383  (5490–16487)  [n=15] | 15562  (11215–22401)  [n=15] | 17279  (10208–23605)  [n=15] | 16175  (12400–  19286)  [n=15] |
| IL-6  (pg/ml) | 0.43  (0–1.11)  [n=77] | 50.64  (23.02–78.65)  [n=81] | 31.33  (17.9–48.98)  [n=81] | 14.59  (9.66–25.98)  [n=74] | 1.59  (1.11–5.39)  [n=15] | 81.43  (33.51–152.95)  [n=15] | 46.35  (22.05–80.32)  [n=15] | 19.93  (15.34–46.23)  [n=15] |

Values are presented as medians (interquartile ranges). Pre, presurgical; POD, postoperative day; pNF-H, phosphorylated neurofilament heavy subunit; PECAM-1, Platelet endothelial cell adhesion molecule-1; MMP-9, matrix metalloproteinase-9; PAI-1, plasminogen activator inhibitor-1; IL-6, interleukin-6. Sample size at each point is indicated in square bracket.

Presurgical values of pNF-H, PAI-1, P-selectin, PECAM-1, and IL-6 were reported in Ref. 12.
