# Supplementary material for: Elevation of serum plasminogen activator inhibitor-1 predicts postoperative delirium independent of neural damage: a sequential analysis
Source: Sci Rep. 2022 Oct 12;12:17091. doi: 10.1038/s41598-022-21682-7 (PMC9556513; doi:10.1038/s41598-022-21682-7)
Supplement: Supplementary file 3 — Supplementary Information 3. [file 41598_2022_21682_MOESM3_ESM.docx]

**Supporting information Table S3** The results of repeated two-way analysis of variance biomarkers for predicting delirium.

|  | **covariate** | | | | | | | |
| --- | --- | --- | --- | --- | --- | --- | --- | --- |
|  | **None** | | | | **Age** | | | |
|  | F-value | *P-*value | partial η2 | observed power | F-value | *P*-value | Partial η2 | observed power |
| **pNF-H** | 0.536 | 0.658 | 0.006 | 0.159 | 0.226 | 0.879 | 0.003 | 0.092 |
| **PAI-1** | 9.437 | < 0.0001 | 0.098 | 0.997 | 13.360 | < 0.0001 | 0.134 | 1.000 |
| **MMP-9** | 4.407 | 0.005 | 0.072 | 0.869 | 4.023 | 0.009 | 0.067 | 0.833 |
| **PECAM-1** | 0.301 | 0.825 | 0.003 | 0.108 | 0.579 | 0.629 | 0.007 | 0.169 |
| **P-selectin** | 2.112 | 0.099 | 0.024 | 0.536 | 2.569 | 0.055 | 0.029 | 0.629 |
| **IL-6** | 0.440 | 0.724 | 0.005 | 0.138 | 1.171 | 0.321 | 0.014 | 0.313 |

pNF-H, phosphorylated neurofilament heavy subunit; PAI-1, plasminogen activator inhibitor-1; MMP-9, matrix metalloproteinase-9; PECAM-1, Platelet endothelial cell adhesion molecule-1; IL-6, interleukin-6.

All the values preoperatively measured were reported in Ref. 12.
