# Supplementary material for: Elevation of serum plasminogen activator inhibitor-1 predicts postoperative delirium independent of neural damage: a sequential analysis
Source: Sci Rep. 2022 Oct 12;12:17091. doi: 10.1038/s41598-022-21682-7 (PMC9556513; doi:10.1038/s41598-022-21682-7)
Supplement: Supplementary file 4 — Supplementary Information 4. [file 41598_2022_21682_MOESM4_ESM.docx]

**Supporting information Table S4** The positivity of pNF-H throughout perioperative periods.

|  | pNF-H positivity | | | |
| --- | --- | --- | --- | --- |
|  | preoperative | POD 1 | POD2 | POD3 |
| PD | 12/15  (80.0%) | 12/15  (80.0%) | 12/15  (80.0%) | 12/15  (80.0%) |
| Non-PD | 33/80  (41.25%) | 35/81  (43.21%) | 37/81  (45.68%) | 37/75  (49.33%) |
| *p* | 0.0058 | 0.0088 | 0.0146 | 0.0295 |

pNF-H, phosphorylated neurofilament heavy chain; POD, postoperative day; PD, postoperative delirium.

The positivity of pNF-H was compared using Pearson’s chi-square test.

The ratio of pNF-H positivity is described in parenthesis.
