# Supplementary material for: Elevation of serum plasminogen activator inhibitor-1 predicts postoperative delirium independent of neural damage: a sequential analysis
Source: Sci Rep. 2022 Oct 12;12:17091. doi: 10.1038/s41598-022-21682-7 (PMC9556513; doi:10.1038/s41598-022-21682-7)
Supplement: Supplementary file 5 — Supplementary Information 5. [file 41598_2022_21682_MOESM5_ESM.docx]

**Supplementary material**: Conditions for marker measurements by enzyme-linked immunosorbent assay or multiplex immunoassay.

|  | dilution | sample volume/well | lower limit of quantification |
| --- | --- | --- | --- |
| pNF-H | 1:3 | 100 mL | 23.5 pg/mL |
| PAI-1 | 1:2 | 50 mL | [pre,2] 17.929pg/mL  [1,3] 17.952 pg/mL |
| PECAM-1 | 1:2 | 50 mL | [pre,2] 1014.361pg/ml  [1,3]1013.660 pg/mL |
| MMP-9 | 1:2 | 50 mL | [pre,2] 113.989pg/mL  [1,3]119.935 pg/mL |
| P-selectin | 1:2 | 50 mL | [pre,2] 224.912pg/mL,  [1,3] 198.207 pg/mL |
| IL-6 | 1:2 | 50 mL | [pre,2] 3.942 pg/mL  [1,3] 3.927 pg/mL |

Limits of quantification were determined using the lowest or highest standard point with a recovery of 70% to 130%. pNF-H, phosphorylated neurofilament-heavy chain; PAI-1, plasminogen activator inhibitor-1;PECAM-1, Platelet endothelial cell adhesion molecule-1; MMP-9, matrix metalloproteinase-9; IL-6, interleukin-6
